# p38α MAPK-mediated induction and interaction of FOXO3a and p53 contribute to the inhibited-growth and induced-apoptosis of human lung adenocarcinoma cells by berberine

**DOI:** 10.1186/1756-9966-33-36

**Published:** 2014-04-26

**Authors:** Fang Zheng, Qin Tang, JingJing Wu, ShunYu Zhao, ZhanYang Liang, Liuning Li, WanYin Wu, Swei Hann

**Affiliations:** 1 Laboratory of Tumor Molecular Biology and Targeted Therapies of Chinese Medicine, 4th Floor, Scientific Research Building, Neihuan West Road No. 55, University City, Panyu District, Guangzhou, Guangdong Province, P. R. China, 510006; 2 Department of Medical Oncology, University of Guangzhou Traditional Chinese Medicine, Guangdong Provincial Hospital of Chinese Medicine, Guangzhou, Guangdong Province, China, 510120

**Keywords:** Berberine, Human lung cancer cells, p38α MAPK, p53, FOXO3a, p21

## Abstract

**Background:**

Berberine (BBR), a component from traditional Chinese medicine, has been shown to possess anti-tumor activity against a wide spectrum of cancer cells including human lung cancer, but the detailed mechanism underlining this has not been well elucidated.

**Methods:**

In this study, the effect of berberine on cell growth and apoptosis were assessed by MTT, flow cytometry and Hoechst 33258 staining assays. The phosphorylation of p38 MAPK and ERK1/2, and expressions of p38 MAPK isoforms α and β, total ERK1/2, p53, FOXO3a and p21 protein were evaluated by Western Blot analysis. Silencing of p38 MAPK isoform α and β, p53, FOXO3a and p21 were performed by siRNA methods. Exogenous expression of FOXO3a was carried out by electroporated transfection assays.

**Results:**

We showed that BBR significantly inhibited growth and induced cell cycle arrest of non small cell lung cancer (NSCLC) cells in the G0/G1 phase in a dose-dependent manner. Furthermore, we found that BBR increased phosphorylation of p38 MAPK and ERK1/2 in a time-dependent and induced protein expression of tumor suppressor p53 and transcription factor FOXO3a in a dose-dependent fashion. The specific inhibitor of p38 MAPK (SB203580), and silencing of p38α MAPK by small interfering RNAs (siRNAs), but not ERK1/2 inhibitor (PD98059) blocked the stimulatory effects of BBR on protein expression of p53 and FOXO3a. Interestingly, inhibition of p53 using one specific inhibitor (Pifithrin-α) and silencing of p53 using siRNAs overcome the inhibitory effect of BBR on cell growth. Silencing of FOXO3a appeared to attenuate the effect of BBR on p53 expression, cell proliferation and apoptosis. Furthermore, BBR induces the protein expression of cell cycle inhibitor p21 (CIP1/WAF1), which was not observed in cells silencing of p53 or FOXO3α gene. Intriguingly, exogenous expression of FOXO3a enhanced the expression of p21 (CIP1/WAF1) and strengthened BBR-induced apoptosis.

**Conclusion:**

Our results show that BBR inhibits proliferation and induces apoptosis of NSCLC cells through activation of p38α MAPK signaling pathway, followed by induction of the protein expression of p53 and FOXO3a. The latter contribute to the BBR-increased p21 (CIP1/WAF1) protein expression. The exogenous FOXO3a, interaction and mutually exclusive events of p53 and FOXO3a augment the overall response of BBR.

## Introduction

Lung cancer is the leading cause of cancer death worldwide with poor 5-year survival rate [1,2]. Current treatments for patients with advanced lung cancer result in rarely curative, and the relapse often occur, which highlights the large need development of novel therapeutic agents against this type of malignancy. Traditional Chinese Medicine (TCM) plays an important role in protecting cancer patients against suffering from complications, assisting in supportive and palliative care by reducing side-effects of conventional treatment and improving quality of life [3] However, the molecular mechanisms by which there herbs in enhancing the therapeutic efficiency against the lung malignancies remain poorly understood.

Berberine (BBR) is a benzylisoquinoline alkaloid extracted from many kinds of medicinal plants that has been extensively used as a TCM and exhibits a wide spectrum of pharmacological activities [4]. Recently, BBR has been reported to have anti-tumor effects on many types of cancer cells [5], and present studies supplied evidence that BBR can inhibit proliferation of non small cell lung cancer (NSCLC) cells, and have no cytotoxic effects on normal human bronchial epithelial cells [6]. However, the detailed mechanism of its anti-cancer activity has not been well elucidated.

The tumor suppressor p53, a sequence-specific transcription factor that activates the expression of genes involved in apoptosis, cell cycle arrest and senescence, has a wide range of functions covering cell cycle control, apoptosis, genome integrity maintenance, metabolism, fertility, cellular reprogramming and autophagy [7-10]. Although different underlying mechanisms for p53 regulation have been proposed for decades, none of them is conclusive. Forkhead homeobox type O3a (FOXO3a, FKHRL1) is also a transcription factor with known tumor suppressor activity and belongs to the family of mammalian forkhead transcription factors, which are regulated by growth factor receptor-induced activation of the phosphatidylinositol 3-kinase (PI3-K)/Akt (or protein kinase B) signaling pathway [11]. Studies in mammalian cells have shown that activation of FOXO3a stimulated the expression of proteins that are involved in apoptosis [11] and cell cycle arrest [12] in different types of cells. FOXO3a was implicated with tumor suppression and inhibition of FOXO3a expression promoted cell transformation, tumor progression and angiogenesis [13]. The cyclin-dependent kinase inhibitors p21 (CIP1/WAF1) has been shown to be involved in the cell cycle control, DNA replication, cell differentiation and apoptosis [14]. Studies demonstrated the link of p53, FOXO3a and p21 signaling in control of cancer cell growth [15-17]. However, the detailed mechanism by these interactions is still inconclusive.

In this report, we show that BBR inhibits growth and induces apoptosis of lung adenocarcinoma cells through activation of p38 mitogen activated protein kinase alpha (p38α MAPK). This, in turn, leads to increase the expressions and protein interactions of p53 and FOXO3a, followed by the induction of cell cycle inhibitor p21 (CIP1/WAF1).

## Materials and methods

### Reagents

Monoclonal antibodies specific for cyclinD1, p38 MAPK isoforms α, extracellular signal-regulated kinase 1/2 (ERK1/2) and their phosphor-forms were purchased from Cell Signaling Technology (Beverly, MA, USA). p38 MAPK isoforms β was ordered from AVIVA System Biology (San Diego, CA, USA). The cyclin D1, p21, p53, FOXO3a and phosphor-form p53 antibodies were abstained from Epitomics (Burlingame, CA, USA). PD98059 (a special inhibitor of ERK1/2), SB203580 (a special inhibitor of p38 MAPK) were purchased from Merck Millipore (Darmstadt, Germany), MTT powder and Pifithrin-α (PFT-α) were purchased from Sigma Aldrich (St. Louis, MO, USA). p38 MAPK isoforms α and β, p53 and FOXO3a small interfering RNAs (siRNAs) were obtained from Santa Cruz (California, CA, USA). Lipofectamine 2000 reagent was purchased from Invitrogen (Carlsbad, CA, USA). The FOXO3a-GFP and N1-GFP plasmids were kindly provided by Dr. Frank M. J. Jacobs (Rudolf Magnus Institute of Neuroscience, University Medical Center, Utrecht, the Netherland) and was reported previously [18]. Berberine was purchased from Chengdu Must Biotechnology (Chengdu, China).

### Cell culture

The NSCLC cell lines (A549, PC9, H1650 and H1299) were obtained from the Chinese Academy of Sciences Cell Bank of Type Culture Collection (Beijing, China) and the Cell Line Bank at the Laboratory Animal Center of Sun Yat-sen University starting March 2012 (Guangzhou, China) and grown in RPMI-1640 medium supplemented with 10% heat-inactivated FBS, HEPES buffer, 50 IU/mL penicillin/streptomycin, and 1 μg amphotericin (complete medium). All cell lines have been tested and authenticated for absence of *Mycoplasma*, genotypes, drug response, and morphology using a commercial available kit (Invitrogen, Shanghai, China) in the Laboratory and in the Animal Center at Sun Yat-sen University. The BBR was dissolved in a small amount of dimethylsulfoxide [DMSO, maximum concentration, 0.1% (v/v)], which was then added to complete cell culture medium prior to addition to sub confluent cells. Cells treated with vehicle only (DMSO, 0.1% in media) was served as control.

### Cell viability assay

NSCLC cells were seeded in 96-well plates at 5 × 10^3^ cells/well, and incubated at 37°C in complete medium for 24 h before the treatment. NSCLC cells were treated with SB203580, PD98059, or pifithrin-α for 2 h or were transfected with control, or p53 and FOXO3a siRNAs for 24 h before exposure of the cells to BBR for an additional 24 h. Afterwards, cell viability were measured using 3-(4,5-dimethylthiazol-2-yl)-2,5-diphenyltetrazolium bromide (MTT) assay according to the instruction from the provider.

### Cell cycle analysis

NSCLC cells were cultured in 6-well plates at 2 × 10^5^ cells/well and treated with increased doses of BBR for 24 h or with SB203580, PD98059, or pifithrin-α for 2 h, followed by BBR for an additional 24 h. Afterwards, the cells were harvested, washed twice with phosphate-buffered saline (PBS), and resuspended in 500 μL of cold PBS and ethanol (1.5 mL) for 2 h at 4°C. The fixed cells were incubated in 1 mL of 0.1% sodium citrate containing propidium iodide (PI) 0.05 mg and 50 μg RNase for 30 min at room temperature (RT) in the dark. The cell cycle analysis was detected by flow cytometry (FC500, Beckman Coulter, FL, USA), and the proportion (percentage) of cells within the G1, S, and G2/M phases of the cell cycle were analyzed using the MultiCycle AV DNA Analysis software (Phoenix Flow Systems).

### Western blot analysis

NSCLC cells were harvested, washed and lysed with 1 × RAPI buffer. Protein concentrations were determined by the Thermo BCA protein assay Kit. Equal amounts of protein from cell lysates were separated on 10% and 12% SDS polyacrylamide gels, and transferred onto polyvinylidene fluoride membranes. Membranes were blocked with 5% non-fat milk in TBST and incubated with primary antibodies against p38 MAPK (including p38 α and β isoforms), ERK1/2 and their phosphor-forms, FOXO3a, p53, p21 and cyclinD1 at 4°C overnight. Afterwards, the membranes were washed and incubated with a secondary antibody against rabbit or mouse IgG conjugated to horseradish peroxidase (Cell Signaling, MA, USA) for 1 h, followed by washing and transferring into ECL solution (Millipore, Darmstadt, Germany), and exposed to X-ray film.

### Treatment with p38 isoforms, p53 and FOXO3a small interfering RNAs (siRNAs)

For the transfection procedure, cells were seeded in 6-well or 96-well culture plates in RPMI 1640 medium containing 10% FBS (no antibodies), grown to 60% confluence, and p38 MAPK isoforms α, β, p53, FOXO3a and control siRNAs were transfected using the lipofectamine 2000 reagent according to the manufacturer’s instructions. Briefly, Lipofectamine 2000 was incubated with Opti-MEM medium (Invitrogen, CA, USA) for 5 min, mixed with siRNA (up to 70 nM), and incubated for 20 min at RT before the mixture was added to cells. After culturing for up to 30 h, the cells were washed and resuspended in fresh media in the presence or absence of BBR for an additional 24 h for all other experiments.

### Cell apoptosis assays

Cell apoptosis was analyzed with Annexin V-FITC/PI Apoptosis Detection Kit (BestBio, Shanghai, China) according to instructions from the manufacturer. Briefly, after treated with BBR for 24 h, the apoptotic cells were harvested by Trypsin (no EDTA) and washed with PBS, then resuspended the cells in 500 μL binding buffer, 5 μL Annexin V-FITC regent and 10 μL PI regents and incubated for 5 min at RT in the dark, followed by detecting cell apoptosis by flow cytometry.

In parallel experiment, Hoechst 33258 staining was used to further analyze cell apoptosis. Cells were cultured in 12-well culture plates and treated with berberine for 24 h. Afterwards, the cells were washed with PBS, and incubated with 500 μL 4% methanal for 10 min, followed by staining with Hoechst 33258 (Sigma, St. Louis, MO, USA) at RT for 10 min, then observed with filters for blue fluorescence under fluorescence microscopy.

### Electroporated transfection assays

NSCLC cells (1 × 10^7^ cells/mL) were washed and centrifuged at 1200 rpm for 5 min, followed by removing the medium and PBS. Afterwards, the cells in the tubes were added Bio-Rad Gene Pulser electroporation buffer. After resuspending the cells, the desired N1-GFP or FoxO3a-GFP plasmid DNA (10 μg/mL) were added and the electroporation plate were put in the MXcell plate chamber and closed the lid in Gene Pulser II Electroporation System (Bio-Rad, CA, USA). The electroporation conditions on the plates to deliver 150 V/5 ms square wave were adjusted until reaching the optimal one. Once the condition has been set and then press “Pulse” to electroporate the cells. After electroporation was completed, the cells were transferred to a tissue culture plate. We typically transfer each 150 μL electroporation sample into a 6-well culture plate containing 2 mL RPMI1640. Cells were incubated for 48 h at 37°C, then treated with BBR for an additional 24 h.

### Statistical analysis

All data were expressed as mean ± SD of three independent experiments, and analyzed by one-way ANOVA followed by post hoc testing or two-way ANOVA followed by Tukey’s Multiple Comparison Test for multiple comparison involved. These analyses were performed using GraphPad Prism software version 5.0 (GraphPad Software, CA, USA). Asterisks showed in the figures indicate significant differences of experimental groups in comparison with the corresponding control condition. P-values <0.05 were considered statistically significant.

## Results

### BBR inhibited human lung carcinoma cell growth and caused G0/G1 arrest in a dose- and time-dependent manner

**Figure 1:**
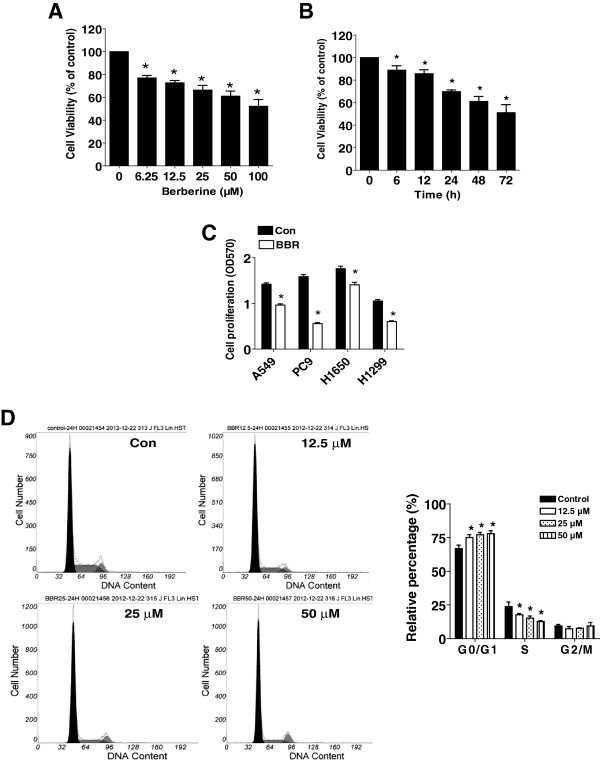
**Berberine (BBR) inhibited human lung carcinoma cell growth and caused G0/G1 arrest in a dose- and time-dependent manner. A**, A549 cells were treated with increased concentrations of BBR for 48 h to examine the cell viability. **B**, A549 cells were treated with BBR (50 μM) for the indicated time to examine the cell viability. **C**, NSCLC cell lines indicated were treated with BBR (50 μM) for 48 h. The cell viability was determined using the MTT assay as described in the Materials and Methods Section and was expressed as percentage of control in the mean ± SD of three separate experiments. *indicates significant difference as compared to the untreated control group (P < 0.05). **D**, A549 cells were treated with increased doses of BBR for 24 h. Afterwards, the cells were collected and processed for analysis of cell cycle distribution by flow cytometry after propidium iodide (PI) staining. And the percentages of the cell population in each phase (G0/G1, S and G2/M) of cell cycle were assessed by Multicycle AV DNA Analysis Software. Data are expressed as a percentage of total cells. Values are given as the mean ± SD from 3 independent experiments performed in triplicate. *indicates significant difference as compared to the untreated control group (P < 0.05).

We first detected the effect of BBR on cell growth in human NSCLC cells A549 by MTT assay. As show in Figure 1A and B, BBR decreased the cell viability in a dose- and time-dependent manner with maximal dose of 50 μM at 48 h treatment. Similar results were also observed in other NSCLC cell lines (Figure 1C). To further examine the effects of BBR on cell proliferation, cell cycle phase distribution of NSCLC cells treated with increased doses of BBR for 24 h was analyzed by Flow cytometry after propidium iodide staining. We showed that, compared with the untreated control cells, BBR significant increased the proportion of cells at G0/G1 phase, while the proportion of cells at S phases were reduced (Figure 1D) suggesting that BBR induced cell cycle arrest in G0/G1 phase in A549 cells.

### BBR induced apoptosis in NSCLC cells

**Figure 2:**
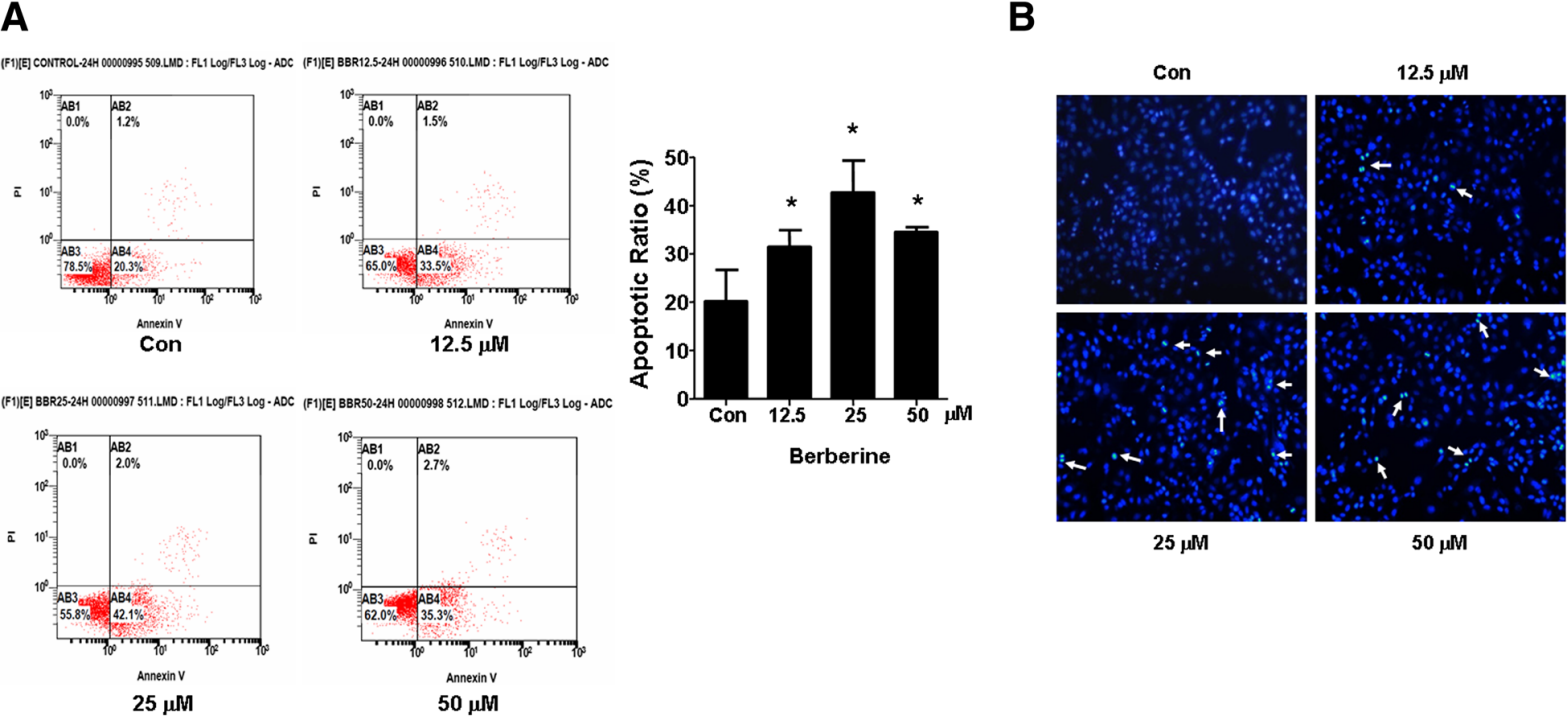
**Berberine induced apoptosis in lung cancer cells. A**, A549 cells were treated with increased concentrations of BBR for 24 h. Afterwards, cells were harvested for analysis of apoptosis using the Annexin V-FITC/PI Apoptosis Detection Kit as detailed in Materials and Methods Section. The AB3 quardrant (annexin V-/PI-), AB4 quadrant (annexin V+/PI-) and AB2 quadrant (annexin V+/PI+) of the histograms indicated the percentage of normal cells, early apoptosis and late apoptosis, respectively. Data are expressed as a percentage of total cells. Values in bar graphs were given as the mean ± SD from three independent experiments performed in triplicate. *indicates significant difference as compared to the untreated control group (P < 0.05). **B**, Apoptotic nuclear morphology changes induced by BBR treatment for 48 h were observed by Hoechst 33258 staining in A549 cells. Panel showed Hoechst 33258 nuclear staining. Arrows indicate chromatin condensation and nuclear fragmentation (×100 magnification). Fluorescence images were taken after Hochest 33258 staining.

We also examine the effect of BBR on apoptosis in NSCLC cells. We found that A549 cells treated with increased concentrations of BBR for 24 h resulted in induction of apoptosis shown in the lower right (AB4) quadrants of the histograms, which were counted as “early” apoptotic cells (Figure 2A) as detected using the Annexin V-FITC/PI stain Apoptotic Detection Kit. After 24 h of treatment, the BBR-induced apoptotic rate was greater than that in the non-treated control cells (Figure 2A). Similar results were obtained in an additional NSCLC cell line PC9 cells (not shown). Meanwhile, the effect of BBA on apoptosis of A549 cells were also tested using Hoechst 33258 staining under fluorescence microscopy. We observed the apoptotic morphologic changes as compared to the control group. The BBR-treated cells showed marked granular apoptotic bodies (Figure 2B). Together, the results above suggested that BBR induced apoptosis in NSCLC cells.

### BBR increased the phosphorylation of p38 MAPK and ERK1/2 in a time-dependent fashion

**Figure 3:**
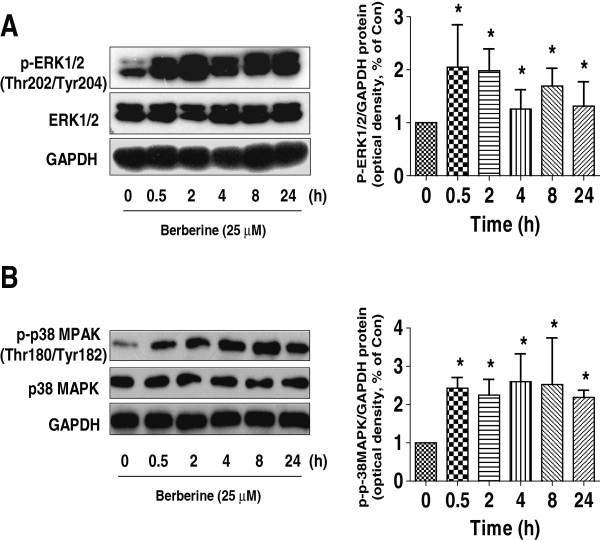
**Berberine increased the phosphorylation of p38 MAPK and ERK1/2 in a time-dependent manner. A-B**, A549 cells were treated with BBR (25 μM) in the indicated times, and cell lysate was harvested and the expression of the phosphorylated or total protein of ERK1/2, p38 MAPK were measured by Western blot analysis using corresponding antibodies. GAPDH was used as loading control. The bar graphs represented the densitometry results of p-ERK **(A)** or p38 MAPK **(B)**/GAPDH as mean ± SD of at least three separate experiments. *indicates significant difference from untreated control cells (P < 0.05).

ERK1/2 and p38 MAPK signaling pathways were involved in apoptosis and cell growth depending on the cell type and stimuli. We showed that BBR increased the phosphorylation of ERK1/2 and p38 MAPK in a time-dependent fashion (Figure 3A-B). Note that the expression of total ERK1/2 and p38 MAPK proteins had no significant changes after BBR treatment. Similar results were obtained in an additional NSCLC cell line PC9 cells (not shown).

### BBR increased protein levels of p53 and FOXO3a through p38 MAPK pathway

**Figure 4:**
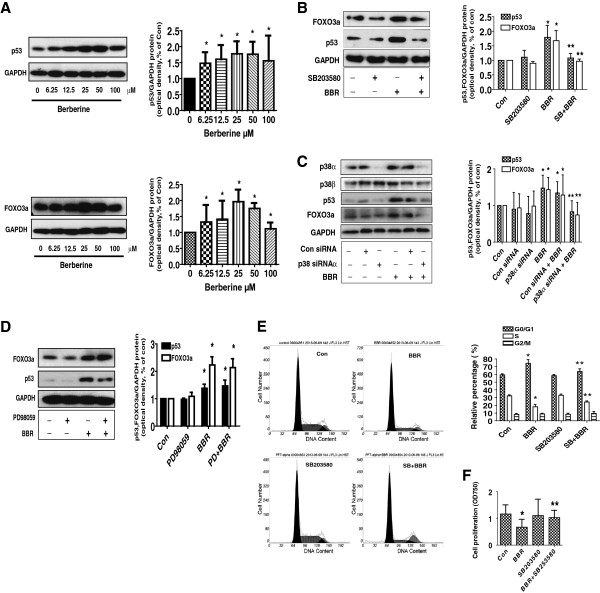
**Berberine increased p53 and FOXO3a protein expression through p38α MAPK pathway. A**, A549 cells were exposed to increased concentration of BBR for 24 h. Afterwards, the expression of FOXO3a and p53 protein were detected by Western blot. **B**-**C**, A549 cells were treated with SB203580 (10 μM) **(B)**, or p38α, β siRNAs (70 nM each) **(C)** for 2 h or 30 h before exposure of the cells to BBR (25 μM) for an additional 24 h. Afterwards, the expression of p38 α or β isoforms, p53 and FOXO3a protein was detected by Western blot. **D**, A549 cells were treated with PD98059 (20 μM) for 2 h and 30 before exposure of the cells to BBR (25 μM) for an additional 24 h. Afterwards, the expression of p53 and FOXO3a protein was detected by Western blot. The bar graphs represent the mean ± SD of p53/GAPDH and FOXO3a/GAPDH of three independent experiments. **E-F**, A549 cells were treated with SB203580 (10 μM) for 2 h before exposure of the cells to BBR (25 μM) for an additional 24 h. Afterwards, the cells were collected and processed for analysis of cell cycle distribution by Flow cytometry after propidium iodide (PI) staining **(E)**. And the percentages of the cell population in each phase (G0/G1, S and G2/M) of cell cycle were assessed by Multicycle AV DNA Analysis Software. Data are expressed as a percentage of total cells. Values are given as the mean ± SD of relative percentage of cell cycle phases from 3 independent experiments performed in triplicate. In separated experiment, the cell viability was determined using the MTT assay **(F)**. *indicates significant difference as compared to the untreated control group (P < 0.05). **Indicates significant difference from BBR treated alone (P < 0.05).

It has reported that p53 cooperated with BBR-induced growth inhibition and apoptosis of NSCLC cells [6]. In this study, we showed that BBR increased FOXO3a, a transcription factor with known tumor suppressor activity [11], protein expression in a dose-dependent manner (Figure 4A). Similar results were obtained with PC9 cells (not shown). Next, we used special inhibitors of p38 MAPK and ERK1/2 to pre-treated A549 cells to examine the role of these kinases in mediating the effect of BBR on induction of p53 and FOXO3a. As shown in Figure 4B, we found that the inhibitor of p38 MAPK (SB203580) abrogated BBR-induced p53 and FOXO3a protein expression, while the inhibitors of ERK1/2 (PD98059) had no effect (Figure 4D). Similar results were observed using p38 MAPK siRNAs; intriguingly, we found that silencing of p38α (Figure 4C), but not p38β isoforms (not shown), abrogated the effect of BBR on p53 or FOXO3a protein expression. This result suggested that activation of p38α isoform was involved in the BBR-induced p53 and FOXO3a protein expression; and that activation of ERK1/2 played no role in this process.

Previously, we showed that BBR induced cell cycle arrest in G0/G1 phase. To further examine the role of p38 MAPK activation in this process, we first treated cells with p38 and ERK inhibitors before exposing the cells to BBR. As shown in Figure 4E-F, compared with BBR treated alone, SB203580 blocked the BBR-caused a decrease in the proportion of cells at S phases (E), and cell proliferation (F). This indicated the role of p38 MAPK activation in mediating the effect of BBR on cell cycle arrest. Note that PD98059 had no effect (not shown).

### BBR-induced inhibition of cell growth and induction of apoptosis were dependent on p53 and FOXO3a protein expression, respectively

**Figure 5:**
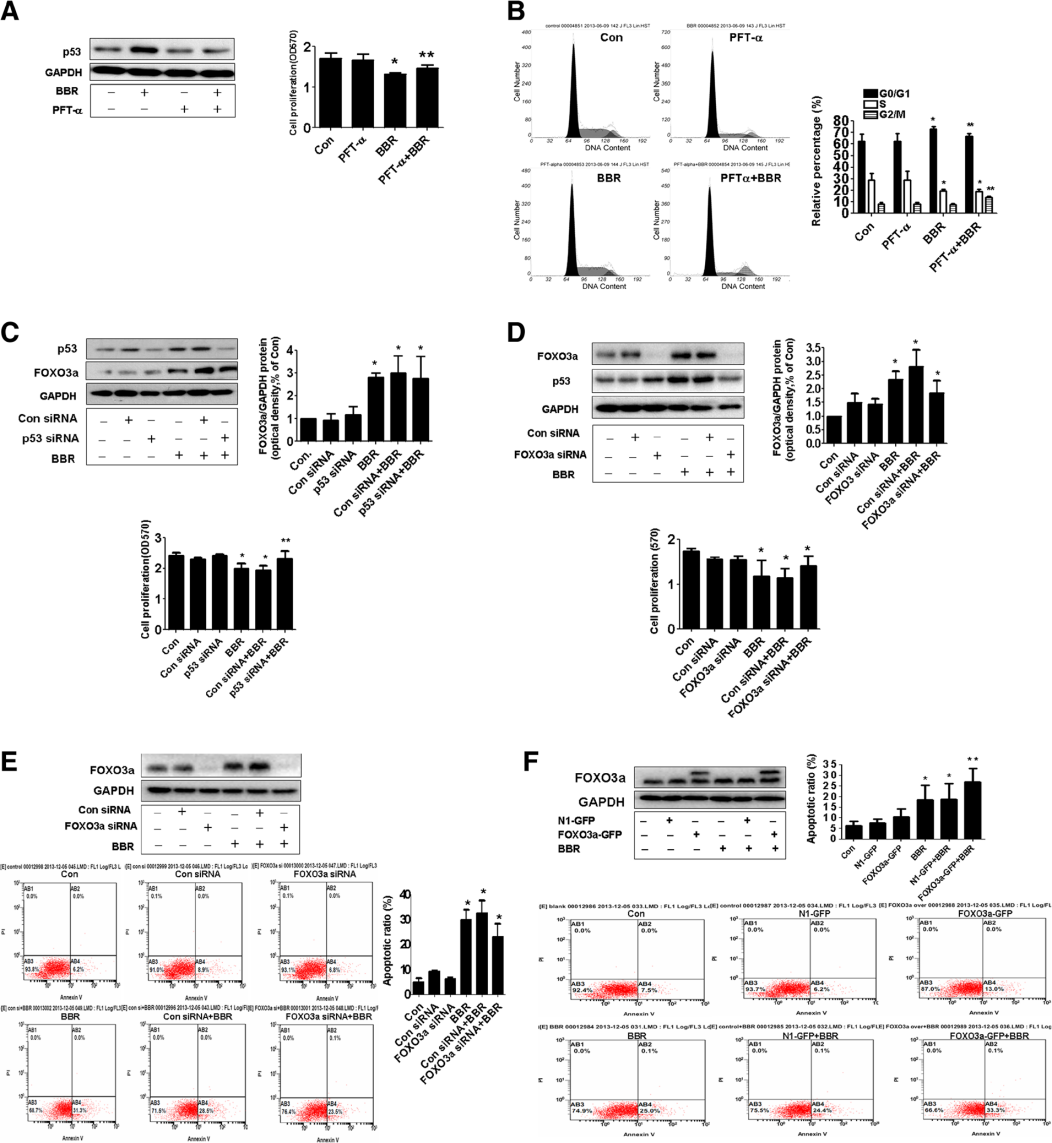
**BBR-induced inhibition of cell growth and induction of apoptosis were dependent on p53 and FOXO3a protein expression in A549 cells. A-B**, A549 cells were treated with Pifithrin-α (10 μM) for 2 h before exposure the cells to BBR (25 μM) for an additional 24 h followed by measuring the p53 protein expression **(A)**. GAPDH was used as internal control **(A)**. And cell cycle was analyzed by flow cytometry after propidium iodide (PI) staining **(B)**. The bar graphs represent the mean ± SD of p53/GAPDH or relative percentage of cell cycle phases of three independent experiments. **C-D**, Cells were transfected with control or p53 or FOXO3a siRNAs with lipofectamine 2000 reagent for 24 h, followed by exposure the cells to BBR (25 μM) for an additional 24 h. Afterwards, the cell proliferation was detected using MTT assays. The expression of p53 and FOXO3a protein was determined by Western blot. The bar graphs represent the mean ± SD of p53/GAPDH and FOXO3a/GAPDH of three independent experiments. **E**, Cells were transfected with control or FOXO3a siRNAs (50 nM each) for 24 h before exposing the cell to BBR for an additional 24 h. Afterwards, cell apoptosis was determined by flow cytometry assays. **F**, Cells were transfected with control (pEGFP-N1) or FOXO3a expression vector (FOXO3a-pEGFP) for 24 h before exposing the cells to BBR for an additional 24 h. Afterwards, the expression of FOXO3a protein and apoptosis were detected by Western blot and flow cytometry, respectively. Data are expressed as a percentage of total cells. Values in bar graphs were given as the mean ± SD from three independent experiments performed in triplicate. *indicates significant difference as compared to the untreated control group (P < 0.05). **Indicates significant difference from BBR treated alone (P < 0.05).

Studies have shown that p53 and FOXO3a regulated cell growth and apoptosis processes. In this study, we found that p53 special inhibitor pifithrin-α showed to overcome the effect of BBR on cell proliferation and G0/G1 arrest (Figure 5A and B). Note that p53 special inhibitor pifithrin-α blocked the effect of BBR on p53 protein expression (Figure 5A upper panel) and induced G2/M phase (Figure 5B). As expected, silencing of p53 by siRNA significantly reversed the BBR-inhibited cell growth (Figure 5C). While silencing of p53 reduced the p53 protein expression (Figure 5C, upper panel), it had no effect on BBR-induced FOXO3a (Figure 5C). On the other hand, silencing of FOXO3a partially reversed the BBR-induced p53 protein expression and cell proliferation (Figure 5D). Furthermore, it attenuated in part the BBR-induced apoptosis as determined by flow cytometry assays (Figure 5E). On the contrary, exogenous expression of FOXO3a enhanced the effect of BBR on apoptosis (Figure 5F). The above findings suggested that induction and potential cross talk of p53 and FOXO3a contributed to the BBR-inhibited cell growth and -induced apoptosis. This also implied that the inhibition of proliferation could by in part a consequence of increased cell apoptosis or *vise versa.*

### BBR increased p21 protein expression dependent of p53 and FOXO3a in lung cancer cells

**Figure 6:**
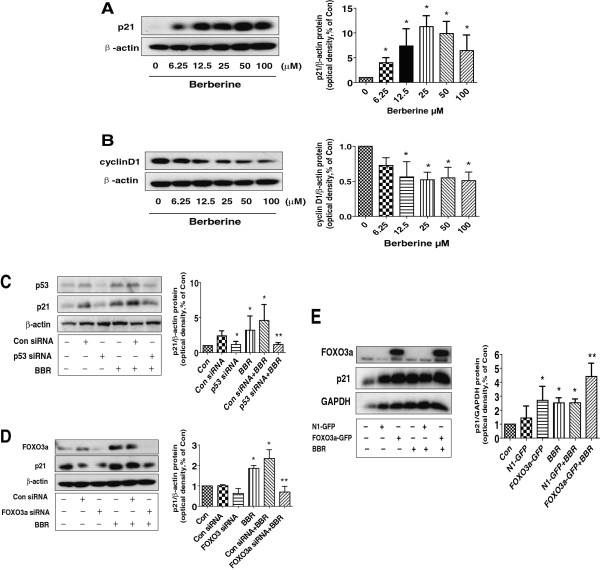
**Berberine increased p21 protein expression through induction of FOXO3a and p53 protein expressions. A-B**, A549 cells were exposed to increased concentration of BBR for 24 h, followed by measuring the protein expression of p21 and cyclin D1 by Western blot. The bar graphs represent the mean ± SD of p21/β-actin or cyclinD1/β-actin of three independent experiments. **C-D**, A549 cells were transfected with control or p53 or FOXO3a siRNAs (50 nM each) for 24 h prior to exposure of the cells to 25 μM BBR for an additional 24 h. Afterwards, Western blot analysis were used measure the protein levels of p53, FOXO3a and p21 using corresponding antibodies. **E**, Cells were transfected with control (pEGFP-N1) or FOXO3a expression vector (FOXO3a-pEGFP) for 24 h before exposing the cells to BBR for an additional 24 h. Afterwards, the expression of p21 protein was detected by Western blot. The bar graphs represent the mean ± SD of p21/β-actin of three independent experiments. *indicates significant difference from control (P < 0.05).

In order to further explore the mechanism by which BBR control cell growth, we tested the cell cycle related protein expression affected by BBR. We found that BBR induced p21 and decreased cyclin D1 expression in a dose-dependent manner with maximal effect at 25 μM (Figure 6A-B). Moreover, we also observed that silencing of p53 or FOXO3a abolished the effect of BBR on p21 (Figure 6C-D) but not cyclin D1 (not shown) protein expression. In addition, the effect of BBR on p21 protein expression was potentiated by overexpression of FOXO3a (Figure 6E). These results indicated that expression of p53 and FOXO3a were required in mediating the effect of BBR on induction of p21 protein expression in lung cancer cells.

## Discussion

Berberine (BBR), a promising phytochemical drug and isoquinoline alkaloid in nature, has been shown to exhibit anti-proliferation or cytotoxic effects against cancer cells of different origins, especially in lung cancer [19-21]. However, the mechanisms by this drug in control of NSCLC cell growth have not been well elucidated. In this study, we confirmed that BBR inhibited NSCLC cell proliferation and induced apoptosis. Moreover, BBR can arrest cell cycle in G0/G1 phase in A549 cells. The concentrations of BBR used here were consistent with or even lower then those reported by others demonstrating significant growth inhibition in different cell systems [21-23]. We realized that a higher dose was needed to inhibit different cancer cell growth, but this was within the range of those reported by others and showed no toxicity [21,22,24].

Induction of cell cycle arrest and apoptosis is regulated by a large number of molecules. In our study, we found that activation of p38α MAPK, but not ERK1/2, was mediated the effect of BBR on cell cycle arrest and induction of p53 and FOXO3a protein expression. Of notes, we demonstrated the unique role of p38α isoform played in this process, whether other p38 isoforms, such as p38γ or p38δ MAPK were also involved in this response required to be determined in the future studies. Consistent with this, the role of p38 MAPK pathway in mediating the cancer cell growth inhibition and induction of apoptosis has been established and reported [25-27]. The p38 MAPK pathway negatively regulated cell proliferation and tumorigenesis. Inactivation of the p38 pathway enhanced cellular transformation and rendered mice prone to tumor development with concurrent disruption of the induction of senescence. Conversely, persistent activation of p38 inhibited tumorigenesis, suggesting a tumor-suppressing function of the p38 pathway [25]. Our results suggested that activation of p38 MAPK was required in mediating the effect of BBR on induction of tumor suppressors p53 and FOXO3a, and lung cancer cell cycle arrest. Note that activation of ERK/12 by BBR played no role in this process, which were different or even opposite reported by others [28,29]. The discrepancy remained unclear; different cell lines and culture conditions may account for this, which needs to be determined with more experiments in the future. The cross-talk between ERK and p38 signaling pathways was reported in other studies [30,31]. However, in this study we have not observed this link. Thus, more experiments may require to confirm this.

In this study, we demonstrated the important role of tumor suppressor p53 in mediating the effect of BBR on cell proliferation and cell cycle arrest, which were consistent with other studies [24,32] suggesting that a p53-dependent pathway was required in this process. Tumor suppressor p53 plays a significant role in the regulation of cell growth, cell cycle arrest, and apoptosis in various cancers [33,34]. p53 controls both the G2/M and the G1 cell cycle checkpoints and mediates reversible growth arrest in human fibroblasts [35]. Increased expression of wild-type p53 arrested cells late in the G1 stage of the cell cycle by stimulating the synthesis of inhibitors of cyclin-dependent kinase p21 (CIP1/WAF1) [35]. Consistent with this, we found that BBR increased p21 protein expression in human lung cancer A549 cells, which was eliminated (not observed) in cells silencing of p53 gene. This suggested that BBR-induced p21 (CIP1/WAF1) was through p53-dependent pathway. P21 (CIP1/WAF1) is a direct target of p53 [36], thus, p53 mediated induction of p21 (CIP1/WAF1) at least contributed to the inhibitory effect of BBR on cell proliferation and cell cycle arrest.

On the other hand, our results suggested that activation of p38 MAPK mediated the BBR-induced FOXO3a protein expression and the latter also contributed to the BBR-inhibited cell growth and -induced apoptosis. It is possible that the inhibition of proliferation can be in part a consequence of increased cell apoptosis or *vise versa.* The FOXO3a is an important tumor suppressor and is under-expressed in many cancers. There are a number of parallels between FOXO3a and p53, both play a pivotal role in regulating the cellular response to stress and damage signals, inducing cell cycle arrest, apoptosis, and DNA repair [37]. Several studies showed that FOXO3a interacts with p53, and that FOXO3a is a p53 target gene [15,38]. In this study, we demonstrated that the potential interaction and mutually exclusive events of p53 and FOXO3a may contribute to enhance BBR-induced apoptosis and -inhibited cell proliferation. However, the detailed mechanism underlining the regulation of these transcriptional networks in mediating the effect of BBR on the control of lung cancer cell survival needs to be elucidated.

Our results also demonstrated a causative role of FOXO3a in mediated the effect of BBR on p21 (CIP1/WAF1) expression. We showed that the knockdown of FOXO3a blocked, while overexpression of FOXO3a augmented the increase in p21 (CIP1/WAF1) protein expression in BBR-treated cells. These, together with the observation from silencing of p53 experiments indicated that p21 (CIP1/WAF1) is not only the direct target of p53 but also function as FOXO3a downstream effector, which may be through the p53-independent way [17]. p53 and FOXO3a share similar target genes including p21(CIP1/WAF1), FOXO factors bind to the promoter of p21 to induce cell cycle arrest at the G1/S transition [39]. Given the fact that p21 (CIP1/WAF1) is involved in regulation of fundamental cellular processes, such as cell proliferation, differentiation, regulation of gene transcription and apoptosis [40,41]. BBR-induced FOXO3a expression may contribute to induce cell apoptosis, which could be in part a consequence of inhibition of NSCLC cell growth. Of note, the dual function of p21 (Cip1/Waf1) was observed in cancerogenesis. On the one hand, p21 (Cip1/Waf1) acts as a tumor suppressor; on the other hand, it prevents apoptosis and acts as an oncogene [40,42]. Therefore, precise understanding the role of p21 (Cip1/Waf1) and relevant signaling pathways involved would help to develop better cancer-treatment strategies.

Study showed that activation of p38 MAPK reduced protein expression of cyclin D1, another cell cycle regulator [43]. Cyclin D1 actives cyclin dependent kinase 4 and 6 (Cdk4/6) and this active complex is essential for the transition to S-phase and further stimulates cell proliferation [44]. In our study, we showed that BBR decreased the cyclin D1 protein expression, but this was not through the p53- or FOXO3a-dependent pathway, which consistent with other studies [45] although opposite results were observed [12,46]. Thus, more studies are required to elucidate the truly connections and precise mechanism underlining this. In addition, whether the BBR-induced pro-apoptotic signaling by p38 MAPK is also activated and the functions of FOXO3a are regulated by p38 MAPK in cells silencing of p53 need to be determined. This may further elucidate pleiotropic anti-cancer mechanisms of this medicinal phyto-chemical compound.

## Conclusion

In summary, our data demonstrate that BBR inhibits growth and induces cell cycle arrest in G0/G1 phase, and apoptosis in NSCLC cells through p38α MAPK-mediated induction of p53 and FOXO3a, followed by p21 protein expression. Thus, the parallel induction and mutually exclusive interaction of p53 and FOXO3a, which act in concert, contribute to mediate the overall responses of NSCLC cell to BBR.

## Abbreviations

BBR: Berberine; TCM: Traditional Chinese medicine; MTT: 3-(4, 5)-dimethylthiahiazo(-z-y1)-3,5-di-phenytetrazoliumromide; p38 MAPK: P38 mitogen activated protein kinases; ERK1/2: Extracellular signal-regulated kinase 1/2; siRNA: Small interfering RNA; NSCLC: Non small lung carcinoma; FOXO3a: Forkhead box-O 3a; PI3-K: Phosphatidylinositol 3-kinase; DMSO: Dimethylsulfoxide; PBS: Phosphate-buffered saline; RT: Room temperature; Cdk4/6: Cyclin dependent kinase 4 and 6; PI: Propidium iodide.

## Competing interests

The authors declare that they have no competing interests.

## Authors’ contributions

SH is fully responsible for the study designing, experiment adjustment and drafting the manuscript. FZ performed most of the experiments involved. QT carried out transfection assays and some protein measurement by Western blot and statistical analysis. SYZ conducted the densitometry, statistical analysis and participated in coordination manuscript. JJW executed the MTT assays, FOXO3a overexpression experiments and statistical analysis. ZYL fulfilled MTT and Western Blot analysis. LLL and WYW coordinated and provided important suggestions including some agents, and critical read the manuscript. All authors read and approved the final manuscript.
